# Transcriptional control of ribosome biogenesis in yeast: links to growth and stress signals

**DOI:** 10.1042/BST20201136

**Published:** 2021-07-09

**Authors:** David Shore, Sevil Zencir, Benjamin Albert

**Affiliations:** Department of Molecular Biology, Institute of Genetics and Genomics of Geneva (iGE3), 30 Quai Ernest-Ansermet, Geneva 4 CH-1211, Switzerland

**Keywords:** gene regulation, growth, proteostasis, ribosome biogenesis, *Saccharomyces cerevisiae* yeast, stress response

## Abstract

Ribosome biogenesis requires prodigious transcriptional output in rapidly growing yeast cells and is highly regulated in response to both growth and stress signals. This minireview focuses on recent developments in our understanding of this regulatory process, with an emphasis on the 138 ribosomal protein genes (RPGs) themselves and a group of >200 ribosome biogenesis (RiBi) genes whose products contribute to assembly but are not part of the ribosome. Expression of most RPGs depends upon Rap1, a pioneer transcription factor (TF) required for the binding of a pair of RPG-specific TFs called Fhl1 and Ifh1. RPG expression is correlated with Ifh1 promoter binding, whereas Rap1 and Fhl1 remain promoter-associated upon stress-induced down regulation. A TF called Sfp1 has also been implicated in RPG regulation, though recent work reveals that its primary function is in activation of RiBi and other growth-related genes. Sfp1 plays an important regulatory role at a small number of RPGs where Rap1–Fhl1–Ifh1 action is subsidiary or non-existent. In addition, nearly half of all RPGs are bound by Hmo1, which either stabilizes or re-configures Fhl1–Ifh1 binding. Recent studies identified the proline rotamase Fpr1, known primarily for its role in rapamycin-mediated inhibition of the TORC1 kinase, as an additional TF at RPG promoters. Fpr1 also affects Fhl1–Ifh1 binding, either independently or in cooperation with Hmo1. Finally, a major recent development was the discovery of a protein homeostasis mechanism driven by unassembled ribosomal proteins, referred to as the Ribosome Assembly Stress Response (RASTR), that controls RPG transcription through the reversible condensation of Ifh1.

## Introduction

Ribosome biogenesis is probably the most energy-intensive anabolic processes carried out in growing cells [1–3]. In rapidly growing budding yeast (*Saccharomyces cerevisiae*) ribosomes are produced at a rate of ∼2000 per minute and are present in ∼200 000 copies per cell. This requires not only the dedication of more than half of all RNAPII initiation events to RP or RiBi genes, but a prodigious output of ribosomal RNA (rRNA), synthesized by the combined action of RNAPI and RNAPIII. The production of RPs and rRNAs is assumed to be highly coordinated in order to ensure rapid and efficient ribosome assembly [2]. At least in yeast, this coordination begins at the level of transcription, which is stimulated under environmental conditions that favor rapid growth and down-regulated under all stress conditions so far examined [1,4]. Here we will focus on recent advances in our understanding of mechanisms underlying RP and RiBi gene regulation, with passing reference to their coordination with rRNA production.

## Three distinct RPG promoter architectures lead to extensive but incomplete co-regulation

**Figure 1.**
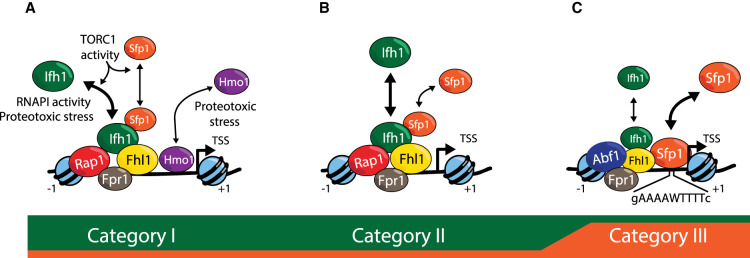
Schematic representation of the three categories of RPG promoter architecture. The color code (below) is indicative of the relative contributions of Ifh1 (green) and Sfp1 (orange) to regulated activation. The regulation of Ifh1 binding by proteotoxic stress and RNAPI activity applies at all categories, as does the regulated binding of both Ifh1 and Sfp1 by TORC1 activity (see text for details). The average position of known transcription factors is shown, together with the stable +1 and −1 nucleosomes. Category I (A) and II (B) promoters have been proposed to harbor two or one unstable (‘fragile’) nucleosomes bound by the chromatin remodeler RSC ([10,88,89]; not shown here) between the indicated +1 and −1 stable nucleosomes, respectively [10,88,89], though this view is controversial [90,91]. Note that the representation of Category III (C) promoters is a composite of various configurations, with Abf1, Fpr1, and Fhl1 present at only a fraction of these promoters, and Rap1 present in place of Abf1 at some. The most ubiquitous feature of Category III promoters would appear to be Sfp1 binding.

Work over many years has revealed a common set of TFs bound to most RPG upstream regulatory regions (hereafter referred to as promoters; [5–16]). The first to be identified was Rap1 (Repressor/activator protein 1), one of a small number of so-called general regulatory factors (GRFs) in yeast [17–21] with features similar to those of mammalian pioneer factors [22]. Although one finds Rap1 at most (127 out of 138) RPG promoters, it binds to an even larger number of other promoters [23]. Instead, the TFs Fhl1 (Forkhead-like 1) and Ifh1 (Interacts with forkhead 1) are equally ubiquitous at RPG promoters but also highly specific for these genes [12,14–16,24,25], with very few non-RPG binding sites. The HMGB protein Hmo1 binds to about one-half of the RPGs bound by Rap1–Fhl1–Ifh1, referred to as Category I genes [5,8,10]. Hmo1 binding co-localizes with that of Fhl1–Ifh1, between Rap1 and the transcription start site (TSS; see Figure 1A), but in addition extends further downstream towards the TSS [13]. Category II is comprised of a roughly equal number of genes, all of which are bound by Rap1, Fhl1, and Ifh1, but not Hmo1 (Figure 1B). At Category I and II genes the relative positions of Rap1 and the Fhl1–Ifh1 pair are strictly conserved, though both are closer to the TSS at Category II genes. Most Category I and II genes also display some detectable level of an additional TF, Split zinc-finger protein 1 (Sfp1) at their promoters. Interestingly, Sfp1 binding is equally prevalent at the large group of RiBi genes implicated in ribosome assembly, as discussed in more detail below [6,11,13,26].

The precise role of Hmo1 at Category I RPG promoters is still controversial. Loss of Hmo1 (*hmo1*Δ) has been reported to abolish both Fhl1 and Ifh1 binding at RPG promoters with only minor effects on transcription or cell growth [5], which led Struhl and colleagues to suggest that proteins other than Fhl1 and Ifh1 might play an important role in RPG activation. However, *fhl1*Δ cells display a severe growth defect and a profound global reduction in both rRNA and mRNA [14], unlike *hmo1*Δ, and *ifh1*Δ is lethal. Furthermore, *hmo1*Δ cells are extremely sensitive to Ifh1 depletion compared to wild-type cells [27], clearly indicating the importance of full Ifh1 function even in the absence of Hmo1. We thus argue that the most parsimonious explanation of these data is that loss of Hmo1 may destabilize or alter Fhl1 and Ifh1 binding in such a way that reduces or abrogates their chromatin immunoprecipitation (ChIP) signal while only modestly affecting their ability to activate transcription. Curiously, the proline isomerase and rapamycin target protein Fpr1 (FK506-sensitive proline rotamase 1), which binds to the majority of RPG promoters (Figure 1C), often co-incident with Rap1 [28], also appears to stabilize Fhl1/Ifh1 binding. Fpr1 is discussed in more detail below.

Regulation of the vast majority of RPGs (the Category I and II genes) is thought to operate largely through mechanisms that control the association of Ifh1 with their promoters, based on the observation that stress (e.g. heat shock or inhibition of the major growth-promoting kinase TORC1 with rapamycin) causes rapid Ifh1 promoter release while leaving both Rap1 and Fhl1 binding unaffected [6,12–15,29]. Anchor-away experiments [30] in which Ifh1 is rapidly depleted from the nucleus in otherwise unstressed cells [31,32] indicate that Ifh1 removal from promoters is by itself sufficient to explain RPG down-regulation under physiological stress conditions. Although Ifh1 is presumed to be a direct activator at RPGs, working through C-terminal activation domains [33], we note that Rap1 itself has been reported to directly activate transcription through interactions either with Taf4, 5, and 12 subunits of TFIID [34,35] or with the Med15 subunit of Mediator [36]. As such, one could imagine that Ifh1 and Rap1 act cooperatively to activate RPGs, or even that Ifh1 works by unmasking or otherwise potentiating Rap1's activation domain. In any event, ChIP analysis points to a major role of Ifh1 in RPG regulation regardless of its mechanism of activation.

Two distinct mechanisms that prevent Ifh1 promoter binding at RPGs under stress conditions have been uncovered. The first to be identified involves a complex containing the ubiquitous casein kinase 2 (CK2), two RiBi proteins (Utp22 and Rrp7) and Ifh1, called CURI [25,37]. The CURI complex titrates Ifh1 away from RPG promoters under conditions where rRNA synthesis is repressed. In cells carrying a mutation in Ifh1 that prevents its association with CURI, or in a strain where RNAPI activity is rendered constitutive through fusion of its Rrn3 and Rpa43 subunits [38], Ifh1 is still rapidly (<5 min) released from RPG promoters following rapamycin treatment but re-binds roughly 20 min later [37]. This finding thus reveals a CURI-dependent long timescale mechanism by which cells can align RPG transcription with that of rRNA but leaves open the question of how Ifh1 is so rapidly removed from RPG promoters in the first place. One attractive hypothesis is that covalent modifications of Ifh1 act to rapidly affect its promoter binding following stress. However, although Ifh1 is modified by both phosphorylation and acetylation [39–43] there is so far no evidence that these modifications are required for its rapid promoter release upon stress. Instead, as will be described below, recent studies indicate that rapid Ifh1 promoter release is provoked by its entrapment in protein aggregates as a consequence of a global impairment of protein homeostasis driven by excess, unassembled RPs [44].

Thirteen of the 138 RPGs, which we have grouped together as Category III genes [10], do not conform to this common promoter architecture and over half of these are instead bound by a different GRF, Abf1, in place of Rap1, and display low levels of both Fhl1 and Ifh1 binding ([10,32,45,46]; Figure 1C). Somewhat surprisingly, the two Category III genes that are bound by Rap1 (*RPL1A* and *RPL18B*), display little or no Fhl1/Ifh1 binding [13,32]. Nearly one-half of Category III genes are also bound by Fpr1, but none by Hmo1. Thus, the Category III genes, though recognized by a similar set of TFs, display a much more heterogeneous promoter architecture. More recent work ([32]; see below) indicates that their most common feature may be direct binding and activation by Sfp1.

Despite these differences in promoter organization, particularly with respect to Category III, all RPGs display remarkably similar up-regulation in response to a glucose pulse or down-regulation in response to two different stress conditions, arrest of secretion by tunicamycin treatment or oxidative stress provoked by diamide addition [32]. Interestingly, though, Category III genes are significantly less down-regulated upon heat shock or when ribosome biogenesis is blocked by the drug diazaborine compared to Category I and II genes [32]. The mechanistic underpinning of these findings and their biological significance will be discussed towards the end of this minireview.

## RPG co-regulation operates through differential deployment of Ifh1 and Sfp1, the primary RiBi gene activator

The observations described above beg the question of what activator(s) might be responsible for regulated activation of Category III RPGs. The roles of Abf1 and Fhl1 were tested by binding site mutagenesis at a limited set of promoters, where mostly modest reductions in expression were observed [45]. Furthermore, Abf1 binding actually appears to increase at these genes following stress [45]. These findings pointed to the existence of an additional key TF at these genes, whose identity was hinted at by the observation that a DNA sequence motif (gAAAATTTTc) bound by Sfp1 *in vitro* [47] is enriched at Category III promoters [32]. Indeed, rapid Sfp1 nuclear depletion leads to strong down-regulation of Category III RPGs, but a much milder effect on both Category I and II genes. Interestingly, just the opposite is true for rapid Ifh1 depletion [32]. Thus, the Category III genes can be considered as a variant of the Category I and II groups in which Sfp1 usurps the primary role in regulated activation normally carried out by Ifh1. Remarkably, expression of Category III genes nevertheless remains tightly coupled to that of the majority of RPGs under most conditions so far examined, with the exception of proteotoxic stress, as discussed below.

Notably, the Sfp1 binding motif is also found at many RiBi gene promoters, and early studies showed that its rapid overexpression via a galactose-inducible promoter fusion leads to a spike in RiBi gene mRNA levels [6,48]. Nevertheless, Sfp1 binding at RiBi gene promoters (as well as at Category III RPGs) is essentially undetectable by ChIP, and the prevalent view was that the RiBi genes are regulated directly through two repressor pathways, one involving Stb3, which binds to a short motif embedded within the conserved RRPE (ribosomal RNA processing element; [29,47,49–51]) and recruits the RPD3-L histone deacetylation complex [52], the other involving the Dot6/Tod6 pair, which achieves the same end through binding to the so-called PAC box (polymerase A and C; [47,52,53]). Sfp1 was thus presumed to act indirectly to promote RiBi gene expression. However, recent application of the chromatin endogenous cleavage-sequencing (ChEC-seq) technique [54] revealed Sfp1's robust association with (RRPE-like) gAAAATTTTc motifs at RiBi gene promoters and those of a large group of additional growth-associated genes [55]. This, and the fact that cytoplasmic anchoring of Sfp1 leads to strong RiBi and RiBi-like gene down-regulation in otherwise unstressed cells, suggests that Sfp1 is a direct activator whose promoter release and re-location to the cytoplasm is sufficient to cause a rapid and strong decrease in transcription of these genes in the absence of active repression pathways [55]. Stb3, and perhaps Dot6/Tod6, may instead be required for the more long-term and complete transcriptional shutdown associated with entry into quiescence [56]. Of note, Stb3 plays a more prominent role in RP, as opposed to RiBi gene repression [52], where its binding motif, but not the more complex RRPE element, is frequently found. Nevertheless, future studies will be required to quantify the relative contribution of activation and repression mechanisms as a function of time during different nutrient- and stress-induced transitions at both RiBi and RP genes.

Expression of several RiBi genes has also been shown to be partly dependent on upstream promoter binding of a GRF (e.g. Abf1, Reb1 or Tbf1; [57]). However, binding of these factors to RiBi gene promoters is not known to be regulated, unlike the case for Sfp1, which is rapidly translocated to the cytoplasm following stress [6,11,26,58]. It will be interesting to determine whether the GRFs act by promoting a chromatin environment more permissive to Sfp1 binding, or instead act in some other, perhaps more direct fashion, to stimulate transcription initiation.

It is worth highlighting here a striking duality in Sfp1's mode of promoter binding, as well as its detection *in vivo*. As mentioned above, Sfp1 binding is detected by ChIP at Category I and II RPGs, though usually with low enrichment, but not at RiBi genes. Conversely, Sfp1 binding is readily detected by ChEC at RiBi genes, as well as an equally large group of growth-associated genes [55], but not at RPGs. This dichotomy almost certainly reflects two distinct modes of Sfp1 promoter binding [55], based upon several observations. First, ChIP-detected Sfp1 binding at Category I and II RPGs, as well as that observed at the promoters of a large group of genes activated during the G1/S cell cycle transition, is not associated with the gAAAATTTTc motif to which Sfp1 binds *in vitro*. Furthermore, Sfp1 binding at Category I and II RPGs is strongly reduced by nuclear depletion of Ifh1. Similarly, Sfp1 binding at promoters in the G1/S regulon is abrogated by depletion of Swi4, a TF specific for these genes. Finally, the importance of the gAAAATTTTc motif for *in vivo* Sfp1 binding, for example at RiBi genes, has been demonstrated recently through endogenous site mutagenesis [59]. Taken together, these findings suggest that Sfp1 is indirectly promoter-bound at RP and G1/S regulon genes, primarily through interactions with other TFs, and possibly independent of a sequence-specific DNA interaction. Why this mode of binding is not detected by ChEC remains unclear, though may be due to its transient nature, since formaldehyde crosslinking renders it ChEC-detectable (B.A., unpublished data). The failure to detect Sfp1 binding at RiBi and Category III RPG promoters by ChIP, even though in these cases its binding would appear to result from a direct interaction with the gAAAATTTTc motif, may be due to poor crosslinking efficiency for A/T relative to G/C base pairs [60].

Finally, we note an additional common feature of RP and RiBi genes, namely their frequent clustering in gene pairs that display increased co-regulation under various stress conditions compared to those genes that are not present in clusters [61–64]. This feature is shared with some other functional groups of genes in yeast [65] and in higher eukaryotes and is proposed to result from enhancer-promoter interactions whose strength as a function of distance scales with genome size [66]. Our improved understanding of the TFs involved in RP and RiBi gene regulation may facilitate the exploration of mechanisms underlying correlated expression of those genes found in close proximity to each other.

## The rapamycin target protein Fpr1 acts as a TF at RPGs

As alluded to above, the yeast FKBP12 prolyl isomerase Fpr1 [67], which also mediates the rapamycin-induced inhibition of TORC1 and interacts physically and genetically with Hmo1, has been identified recently as a novel TF at RPGs [28]. Fpr1 is robustly detected by ChIP at the promoters of most RPGs (95%; thus, not exclusively at those bound by Hmo1) and at very few (∼10) other genes. One common feature of the sites where Fpr1 binding is detected is the presence of Rap1, and deletion of the Rap1 binding site at the *RPL25* promoter does indeed abolish Fpr1 binding there. Nevertheless, there are many other promoters bound by Rap1 where Fpr1 binding is not observed. The determinants of Fpr1 binding specificity thus remain to be unraveled. Interestingly, the rapamycin binding activity of Fpr1, but not its proline isomerase activity, is required for its RPG promoter binding and transcriptional activation. However, genetic studies clearly indicated that Fpr1's effect on RPGs is independent from its interaction with TORC1 ([68]; reviewed in [69]).

The effect of *fpr1*Δ on RPG transcription is less than one might expect given its widespread binding, with only four RPGs showing a significant decrease in steady-state mRNA levels, compared to the ∼50 genes affected by *hmo1*Δ [28]. Interestingly, an *fpr1*Δ *hmo1*Δ double mutant displays a number of additive or synergistic decreases in transcription of specific RPGs, consistent with its severe slow-growth phenotype (or lethality; [67]). Remarkably, *fpr1*Δ *hmo1*Δ can be rescued by an extra gene copy of *RPL25*, whose transcription is strongly impaired in *fpr1*Δ *hmo1*Δ cells [28]. Exactly how Fpr1 contributes to RPG transcription is still unclear but appears to be related to an effect on the Fhl1/Ifh1 activator pair, as is the case for Hmo1. Rapid degradation of Fpr1 via the auxin-induced degron system results in a significant decrease in Fhl1 ChIP-seq signal at many RPGs [28], similar to that reported for *hmo1*Δ. Again, though, the significance of this finding is unclear, since both the transcriptional and growth phenotypes of *fhl1*Δ are much more severe than those of *fpr1*Δ. Furthermore, Ifh1 depletion by anchor-away, which is carried out in a *fpr1*Δ strain background, clearly points to a major role for Ifh1 in both activation and regulation of RPGs that operates independently of Fpr1 [32].

The identification of Fpr1 as a novel TF highlights a persistent mystery regarding the binding specificity of the constellation of TFs found at RPGs. Indeed, only Rap1 binding consistently correlates with the presence of a recognizable binding motif. Fhl1, a forkhead-domain protein, recognizes a specific motif *in vitro* [10,47,70] yet uses this capability to augment its binding at only a small fraction of RPGs [10]. Sfp1, as pointed out above, appears not to use its sequence-specific binding mode at most (Category I and II) RPGs, but instead relies on Ifh1, and perhaps other factors. Hmo1 may rely upon multiple G/C-rich motifs similar to the IFHL motif to generate strong binding at Category I promoters [10,32]. Fpr1, on the other hand, contains no known DNA-binding motif and its high degree of specificity for RPG promoters remains unexplained. Though Rap1 binding may be necessary for association of Fpr1 at RPG promoters [28], it cannot be sufficient given the large number of non-RPG promoters that bind Rap1 but not Fpr1. One is thus left with the impression that the majority of RPG TFs rely upon small networks of cooperative interactions (protein–protein and/or protein–DNA) to achieve specificity. Future studies will clearly be required to resolve this issue.

## Ribosome biogenesis and protein homeostasis are tightly coupled

Previous studies have pointed to links between ribosome biogenesis and protein homeostasis (proteostasis). For example, the primary source of ubiquitin in growing yeast, and probably most metazoan cells, comes in the form of N-terminal protein fusions to two specific ribosomal proteins [71,72], thus directly linking ubiquitin availability to RPG expression. In addition, deletion of *TOM1*, which encodes a E3 ubiquitin ligase primarily dedicated to the degradation of unassembled RPs, profoundly impacts cellular proteostasis [73,74]. Two recent reports [44,75] have now revealed that ribosome biogenesis itself can be a major endogenous source of proteostasis stress, which provokes an adaptive response that we refer to as the ribosome assembly stress response (RASTR). RASTR is a consequence of the frenetic pace of ribosome assembly in rapidly growing cells, which, even in the absence of external stress, generates a pool of unassembled (‘orphan’) RPs that is sensed by the cellular protein homeostasis system. Numerous chemical and genetic perturbations of ribosome assembly only serve to augment the level of aggregation-prone orphan RPs and reveal a highly specific effect on transcription by RNAPII: up-regulation of heat shock factor 1 (Hsf1) target genes and down-regulation of RPGs. Activation of Hsf1 is probably due to relief of negative regulation by the Hsp70 proteins Ssa1/2, perhaps through effects on the essential Hsp40 co-chaperone Sis1 [76,77]. RP down-regulation, on the other hand, turns out to result from the rapid release of Ifh1 from RPG promoters, coincident with its accumulation in nuclear aggregates [44].

Remarkably, then, excess unassembled RPs negatively regulate the genes that encode for them through sequestration of their key transcriptional activator, Ifh1. Significantly, Ifh1 appears to be largely composed of intrinsically disordered domains (IDRs; http://original.disprot.org/metapredictor.php), a common feature of proteins capable of forming liquid–liquid phase separated condensates [78]. Although the nature and underlying mechanisms driving Ifh1 condensation are unknown, it is interesting to note that Ifh1 contains highly acidic regions that might promote its aggregation with basic IDRs of orphan RPs [79]. It will clearly be important to identify regions of Ifh1 required for its regulated condensation as well as the possible involvement of covalent modification (e.g. phosphorylation [80] and acetylation) in controlling this process.

**Figure 2.**
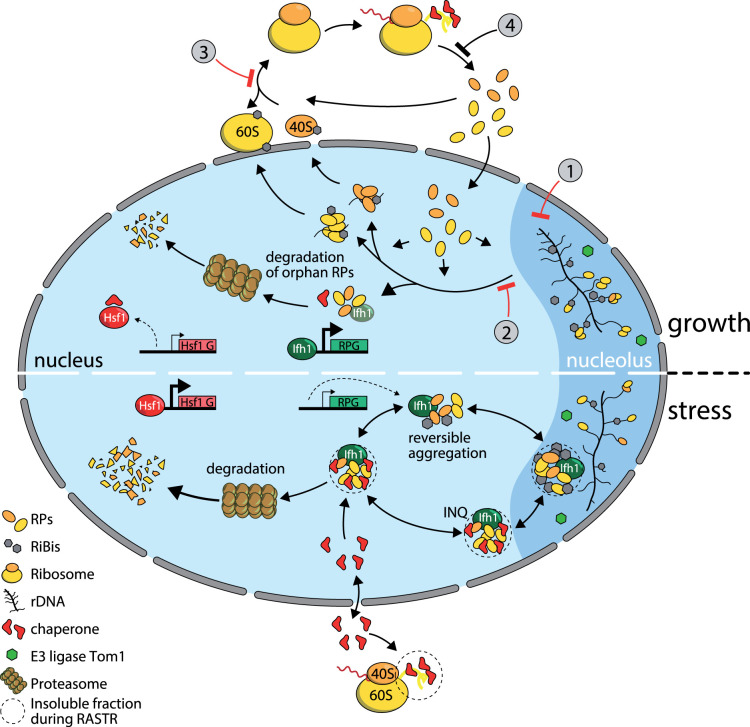
Schematic view of the ribosome biogenesis — proteostasis connection. The top half indicates flow through the system under normal growth, the bottom under RASTR conditions. Important elements not directly labeled are indicated in the key (bottom left). Numbers indicate steps that have been blocked by genetic or chemical perturbations [44,75]: (1) rRNA transcription by Top1 degradation; (2) ribosome assembly by Utp8 or Utp13 degradation; (3) ribosome assembly by diazaborine treatment; (4) RP translation by cycloheximide treatment. A specific block of RPG transcription by Ifh1 anchor-away (not indicated here) dampens Hsf1 activation in otherwise wild-type conditions or in combination with RASTR activation [44]. The presence of Ifh1 in nucleolar, INQ (intranuclear quality control compartment; [92,93]) and nuclear aggregates containing RPs is based upon preliminary observations (B. A., unpublished results) and should be considered speculative at this point. Similarly, the nucleolar localization of the E3 enzyme Tom1 is supported by unpublished observations (https://yeastgfp.yeastgenome.org/displayLocImage.-php?loc=22199 and B.A.).

This transcriptional arm of RASTR appears to be a highly adaptive response since most Hsf1 target genes encode for chaperones of proteasome components and the Ifh1 activator is quite specific for RPGs, whose products are responsible for generating the stress in the first place. It is worth pointing out here that RASTR is accompanied not only by reduced solubility of RPs and Ifh1, but by condensation of a significant number of additional proteins, many of which are involved in ribosome assembly and translation [44,75]. A schematic depiction of some of the key features of RASTR is presented in Figure 2.

The notion that RASTR operates even in unstressed rapidly growing cells follows from the observation that treatment of cells with cycloheximide provokes an opposite transcriptional response: up-regulation of RPGs and down-regulation of Hsf1 target genes, suggesting that even under these conditions Ifh1 activity is mildly suppressed and Hsf1 partially actived. In fact, RASTR itself requires ongoing protein synthesis and is attenuated by cycloheximide treatment or by specific reduction of RPG transcription by nuclear depletion of Ifh1, even in cells where ribosome assembly is simultaneously perturbed [44]. Remarkably, the cycloheximide-sensitive transcriptional response characteristic of RASTR is observed in other stress conditions, such as heat shock or TORC1 inhibition by rapamycin treatment [44]. This may be a consequence of the rapid inhibition of RNAPI activity and arrest in rRNA production under a wide variety of stress conditions, which may quickly lead to a spike in unassembled RPs, should their translation continue more or less unabated. The observation that RASTR is induced by different stresses not obviously associated with ribosome assembly raises the question of whether Ifh1 sequestration in dynamic condensates is the sole mechanism conferring rapid RPG down-regulation in response to stress. As mentioned above, Ifh1 is subject to extensive covalent modification, for example by CK2 phosphorylation, which influences its interaction with the Fhl1 FHA domain [37,42], and through acetylation by the SAGA-associated Gcn5 lysine acetyltransferase enzyme, which both stabilizes Ifh1 and acts to dampen its activation function following a glucose pulse [39,40]. Furthermore, PKA-dependent phosphorylation of Ifh1 acts to limit replicative lifespan with no apparent effect on RPG transcription rates [39]. Mutations that abrogate these and other modifications have yet to be tested explicitly for effects on rapid Ifh1 promoter release during stress.

We recently found [32] that proteotoxic stress (induced either by heat shock or perturbation of ribosome biogenesis by rapid topoisomerase depletion) also causes the release of Hmo1 from Category I RPG promoters. Whether this contributes to their down-regulation under these conditions is not yet known but would appear to be unlikely since Hmo1 is not released from these promoters following rapamycin treatment [32], which also causes their strong and rapid down-regulation. As predicted by the minimal or non-existent role of Ifh1 at Category III promoters, they are significantly less down-regulated by RASTR compared to both Category I and II genes. Interestingly, a large number of Category III RPs have known dedicated chaperones, [81] which would in principle reduce their need for transcriptional down-regulation.

In summary, the two new studies outlined above [44,75] illuminate a regulatory mechanism in yeast that senses the quality of ribosome assembly, at least in part through the detection of unassembled orphan RPs and uses this information to restore protein homeostasis through specific transcriptional and post-transcriptional regulation. RASTR would appear to be a more evolutionarily ancient protein homeostasis mechanism than the well characterized p53-MDM2 system that monitors unassembled RPs. Although the conservation of RASTR in metazoans has yet to be explicitly addressed, several studies hint at its possible involvement in ribosomopathies [82,83] and cancer [84–87].

## Perspectives

- Ribosome biogenesis is a remarkably complex and energy-intensive process essential for rapid cell growth. The requirement to coordinate the production and assembly of nearly 80 ribosomal proteins with ribosomal RNA and to tune this process in response to fluctuating environmental conditions, imposes unique regulatory challenges, which are solved in yeast by the combinatorial action of multiple transcription factors deployed in more than three distinct configurations.
- Recent studies reveal that perturbations to ribosome biogenesis leading to an excess of unassembled ribosomal proteins induce a global yet highly focused protein homeostasis reaction, the ribosome assembly stress response. This multipronged regulatory system acts at transcriptional and post-transcriptional levels, via still poorly understood protein condensation mechanisms, to help restore cellular proteostasis.
- Future studies will explore the underlying molecular mechanisms that drive RASTR in yeast and provide a clearer understanding of how this response improves cell fitness under stress. We anticipate that new studies in metazoan systems will reveal the extent to which this system is evolutionarily conserved and its potential implication in development and disease, specifically in ribosomopathies but more generally in cancers, where ribosome biogenesis underlies rapid and uncontrolled proliferation.

## Abbreviations

Abf1: ARS binding factor 1
CK2: casein kinase 2
CURI: CK2/Utp22/Rrp7/Ifh1
FHA: forkhead-associated
Fhl1: forkhead-like 1
Fpr1: FK506-sensitive proline rotamase 1
GRF: General Regulatory Factor
Hmo1: high mobility group 1
Ifh1: interacts with forkhead 1
INQ: intranuclear quality control compartment
Rap1: repressor/activator protein 1
RASTR: ribosome assembly stress response
RiBi: ribosome biogenesis
RNAPI: RNA polymerase I
RNAPII: RNA polymerase II
RNAPIII: RNA polymerase III
RP: ribosomal protein
RPG: ribosomal protein gene
rRNA: ribosomal RNA
Sfp1: split zinc-finger protein 1
TF: transcription factor
TSS: transcription start site
